# New Appliances and Things Medical

**Published:** 1903-04-25

**Authors:** 


### NEW APPLIANCES AND THINGS MEDICAL.

**[We shall be glad to receive at our Office, 28 & 29 Southampton Street, Strand, London, W.C., from the manufacturers, specimens of all new preparations and appliances which may be brought out from time to time.]**

### ICILMA CASTILE SOAP AND FLUOR CREAM.

(Icilma Company, 142 Gray's Inn Road, London, W.C.)

The Icilma preparations are claimed to be prepared from the water which is obtained at the Silama Springs—a water which contains silica in solution. Silica has certain medicinal properties which are of value in maintaining the nutrition of the skin. Icilma Castile soap is to be recommended for toilet use; it is an example sample of a Castile soap, and may be used with safety and advantage for ordinary toilet purposes, or medicinally when the skin is subject to inflammatory conditions. Icilma fluor cream, which is soluble in water and of soothing properties may be used under similar conditions.

### WEST INDIAN TREACLE.

(Fowler, Limited, Glasshouse Wharf, Blackwall, London, E.)

The elaborately refined golden syrups to which we are now accustomed are very different in appearance to the old-fashioned green treacles of our youth. From a gastronomic and dietetic point of view it is questionable whether this high degree of refining has any advantage, and, further, it is to say the least doubtful whether we are not simply buying glucose under another name, when we procure these wonderfully brilliant liquid sugars sold under the name of golden syrups. We have recently examined a specimen of Fowler's West India treacle. It is guaranteed to be the product of the cane which is grown in the West Indies. It is a fine example of an old-fashioned green treacle, with the characteristic odour. For cooking purposes, as for instance in the making of ginger-bread and puddings, it is infinitely superior to the more refined golden syrups, and for sweetening porridge or eating on bread and butter, most people would prefer it to many of the treacles which are more elegant in appearance.

### THE QUICKLIT FIRE-LIGHTER.

(The British Fire-Lighter Company, Limited, Belvedere, Kent.)

These new fire-lighters are composed of well dried wood containing a good proportion of resinous oils. They are square in shape, and consist of one piece of wood sawn in various directions, so that a good draught can be maintained. We have made a practical trial of them and find that they are ideal fire-lighters, rapid, reliable, and cheap.

